# Renal Involvement in Cancer Patients Undergoing Oncology Therapies: Implications for Personalized Treatment Strategies

**DOI:** 10.3390/jpm16030163

**Published:** 2026-03-15

**Authors:** Silvia Lai, Alessandra Punzo, Adolfo M. Perrotta, Giuseppe Guaglianone, Silverio Rotondi, Paolo Menè, Paolo Izzo, Sara Izzo, Andrea Polistena, Lida Tartaglione, Francesca Tinti, Marta Barattini, Andrea Botticelli, Simone Scagnoli, Daniele Santini, Anna P. Mittherhofer, Giovanni Pintus

**Affiliations:** 1 Department of Translational and Precision Medicine, Nephrology Unit, Policlinico Umberto I University Hospital, Sapienza University of Rome, 00185 Rome, Italybarattini.marta@gmail.com (M.B.); 2 Department of Hospital Pharmacy, “Azienda Sanitaria Locale (ASL) Roma 4”, 00053 Civitavecchia, Italy; 3 Department of Clinical and Molecular Medicine, Nephrology and Dialysis Unit, St. Andrea University Hospital, Sapienza University of Rome, 00185 Rome, Italy; 4 Pietro Valdoni” Department of Surgery, Policlinico “Umberto I”, Sapienza University of Rome, 00185 Rome, Italyandrea.polistena@uniroma1.it (A.P.); 5 Plastic and Reconstructive Surgery Unit, Multidisciplinary Department of Medical-Surgical and Dental Specialties, Università degli Studi della Campania “L.Vanvitelli”, 80138 Naples, Italy; 6 Department of Radiological Sciences, Oncology and Pathology, Sapienza University of Rome, 00185 Rome, Italy; andrea.botticelli@uniroma1.it (A.B.); simone.scagnoli@uniroma1.it (S.S.); daniele.santini@uniroma1.it (D.S.); 7 Department of Systems Medicine, Nephrology and Dialysis Unit, University of Rome Tor Vergata, 00133 Rome, Italy; annapaola.mitter@uniroma2.it (A.P.M.);

**Keywords:** renal toxicity, acute kidney injury, chronic kidney disease, immunotherapy, immune-related adverse events, cancer treatment, renal monitoring, precision oncology, personalized medicine

## Abstract

**Introduction:** Oncological therapies have significantly improved patient outcomes but are increasingly associated with renal toxicity, which can markedly influence therapeutic decisions. Integrating early identification of kidney injury into clinical workflows is essential for personalized medicine, allowing treatment tailoring based on individual risk profiles. **Aim:** To evaluate the incidence of acute kidney injury (AKI) and chronic kidney Disease (CKD); assess indices of renal function recovery in patients who developed AKI; and investigate the incidence of renal immune-related adverse events (irAEs) in patients receiving immunotherapy. **Materials:** Renal function, serum electrolytes, inflammatory markers, blood gas analysis, and urinalysis were evaluated at baseline before oncological therapy (T0), after approximately 2 weeks (T1), and after 3 months (T2). **Results:** Seventy patients were analyzed (median age 71.5 years). AKI occurred in 43 patients (61.4%) and CKD in 18 (25.7%). Patients receiving immunotherapy displayed significantly higher blood urea nitrogen (*p* < 0.01) and creatinine (*p* < 0.01) levels compared to those undergoing traditional therapies (targeted therapy and chemotherapy). Treatment discontinuation was required in 14 (56%) immunotherapy patients versus 7 (19.4%) receiving traditional therapy (anti-VEGF and cisplatin) (*p* < 0.01). Among 25 immunotherapy-treated patients, 13 (52%) developed immune-related adverse events (irAEs). Patients with irAEs predominantly experienced AKI (92.3%), whereas those without irAEs showed both AKI and CKD (44.4%) (*p* < 0.01). Treatment discontinuation occurred in 84.6% of patients with irAEs compared to 11.1% without irAEs (*p* < 0.001). **Conclusions:** We showed a high incidence of AKI and CKD among cancer patients; in particular, the majority of patients receiving immunotherapy presented irAEs. CKD also occurs in association with comorbidities, such as previous use of NSAIDs, investigations with contrast agents and episodes of AKI on CKD determined by drugs. It seems necessary for there to be multidisciplinary collaboration between oncologists and nephrologists to individualize treatment plans; thus allowing the non-suspension of therapy, which positively influences the prognosis of patients.

## 1. Introduction

Cancer is the second leading cause of morbidity and mortality in Europe, accounting for approximately 3.7 million new cases per year, with an average age at diagnosis of around 65 years [1]. Modern oncological treatment includes a broad range of therapeutic strategies such as conventional chemotherapy (e.g., irinotecan, capecitabine, oxaliplatin, gefitinib, erlotinib, gemcitabine, cisplatin, paclitaxel, carboplatin, docetaxel, vinorelbine, topotecan, etoposide); targeted therapies aimed at growth factors and their receptors, including anti-angiogenic agents such as vascular endothelial growth factor (VEGF) inhibitors (e.g., bevacizumab, ramucirumab) and inhibitors of cell-cycle or enzymatic pathways; and immunotherapy, including immune checkpoint inhibitors (ICIs) (e.g., ipilimumab, pembrolizumab, nivolumab, atezolizumab), cancer vaccines, adoptive cell therapy, chimeric antigen receptor T cells (CAR-T), and monoclonal antibodies used alone or in combination. Although these innovations have significantly improved cancer outcomes, they may also lead to acute kidney injury [AKI] and chronic kidney disease (CKD) [2].

Some malignancies exert direct nephrotoxic effects—such as multiple myeloma, neoplastic infiltration, or paraneoplastic syndromes. Cancer may affect the kidneys through glomerular injury or toxic effects of medications or radiation, leading to acute conditions (e.g., thrombotic microangiopathy, AKI, or interstitial nephropathies) or chronic disorders (e.g., CKD progression after nephrectomy for renal cancer, interstitial fibrosis, or electrolyte disturbances) [3]. Antineoplastic agents can damage any part of the nephron, resulting in clinical manifestations such as proteinuria, hypertension, electrolyte abnormalities, glomerulonephritis, acute and chronic interstitial nephritis, and AKI. In particular, anti-VEGF agents can induce renal injury primarily through endothelial dysfunction and disruption of glomerular capillary integrity, leading to proteinuria, hypertension, and thrombotic microangiopathy. In contrast, cytotoxic chemotherapies, such as cisplatin, cause direct tubular toxicity, characterized by proximal tubular cell injury, oxidative stress, and inflammatory responses, which may result in AKI. Immunotherapy may also trigger a broad spectrum of immune-related adverse events (irAEs), including renal irAEs. These events arise when immune activation induced by ICIs inadvertently targets healthy kidney tissue, most commonly causing acute tubulointerstitial nephritis or immune-mediated glomerular diseases [4]. Current international guidelines recommend prompt recognition, grading, and a stepwise immunosuppressive approach. For grade 1 irAEs, ICIs can generally be continued with close monitoring, except for selected neurologic, hematologic, and cardiac toxicities. For grade 2 toxicities, ICIs should usually be withheld and corticosteroid therapy initiated (prednisone 0.5–1 mg/kg/day or equivalent). For grade ≥ 3 irAEs, ICIs should be discontinued, and high-dose corticosteroids (prednisone or methylprednisolone 1–2 mg/kg/day) are recommended. If there is no clinical improvement within 48–72 h, second-line immunosuppression, depending on the organ involved, should be initiated and/or dialysis [5]. Improved supportive care, the increasing number of elderly patients with comorbidities, exposure to multiple chemotherapeutic regimens, repeated contrast-enhanced CT imaging, and the introduction of new therapeutic strategies have contributed to a rise in patients at risk of AKI and CKD [2]. Moreover, the presence of pre-existing CKD, AKI, or other renal disorders may significantly influence treatment choices and patient outcomes in oncology [6,7].

Aim of the study: to evaluate the incidence of AKI and CKD; assess indices of renal function recovery in patients who developed AKI; and investigate the incidence of irAEs in patients receiving immunotherapy.

## 2. Materials and Methods

### 2.1. Study Design and Subjects

We conducted an observational, longitudinal, single-center cohort study involving adult patients with oncological disease followed at the oncology and nephrology outpatient clinics of Policlinico Umberto I between July 2024 and January 2025. Clinical (body mass index, smoking status, arterial pressure and heart rate) and laboratory parameters were assessed at baseline prior to oncologic therapy (chemotherapy, targeted therapy and immunotherapy) (T0), after approximately two weeks (T1), and after three months (T2). Variables were predefined as either analytical variables or descriptive clinical variables. Exclusion criteria included pediatric patients, individuals with hematological malignancies, and patients undergoing renal replacement therapy and/or kidney transplantation. AKI and CKD were defined according to KDIGO criteria. AKI was diagnosed based on changes in serum creatinine or urine output (KDIGO 2012). CKD was defined as eGFR < 60 mL/min/1.73 m^2^ and/or markers of kidney damage persisting ≥ 3 months (KDIGO 2024) [8,9]. In participants with pre-existing CKD, AKI was defined in accordance with the KDIGO criteria, using changes in serum creatinine relative to the individual baseline value. AKI was diagnosed as an absolute increase in serum creatinine of ≥0.3 mg/dL within 48 h or a relative increase of ≥50% from baseline within 7 days. Baseline kidney function was defined as the most recent stable outpatient serum creatinine measurement obtained prior to study inclusion. When available, urine output criteria (<0.5 mL/kg/h for ≥6 h) were also applied [10]. AKI severity was staged according to KDIGO guidelines. In patients with underlying CKD who developed AKI during the study period, management followed a standardized, guideline-based approach. This included systematic evaluation for pre-renal, intrinsic, and post-renal causes of kidney injury; prompt correction of hemodynamic disturbances; optimization of volume status; and discontinuation or dose adjustment of potentially nephrotoxic medications based on eGFR. Renal function and electrolyte levels were monitored serially throughout the AKI episode [11]. According to international consensus in AKI research, including interpretations of KDIGO criteria, renal recovery is defined as the return of kidney function toward baseline—reflected by normalization or significant improvement in serum creatinine and eGFR—and independence from renal replacement therapy when previously required [12]. The study protocol was approved by the Clinical Research Ethics Committee of Sapienza, University of Rome, Italy, and Policlinico Umberto I hospital with Rif. 7641 and Prot. 0716/2024.

### 2.2. Laboratory Measurements

The following laboratory tests were performed using standard methodologies: serum glucose (mmol/L), creatinine (mg/dL), blood urea nitrogen (mg/dL), serum electrolytes (mEq/L), serum uric acid (mg/dL), lactate dehydrogenase (LDH) (mg/dL), serum protein electrophoresis, international normalized ratio (INR), C-reactive protein (μg/L), prothrombin time (PT) (sec), activated partial thromboplastin time (aPTT) (sec), D-dimer (ng/mL), hemoglobin (g/dL), and white blood cell count (10^3^/mL). Arterial blood gas analysis, urinalysis, and the neutrophil-to-lymphocyte ratio (NLR) were also evaluated. Estimated glomerular filtration rate (eGFR) was calculated using the Modification of Diet in Renal Disease (MDRD) equation. Twenty-four-hour urinary protein excretion (g/24 h) was also measured. Variables were predefined as either analytical variables or descriptive clinical variables.

### 2.3. Statistical Analysis

Statistical analyses were performed using SPSS software, version 26.0. Data distribution was assessed using the kurtosis coefficient. Results are expressed as median and interquartile range (IQR) or as absolute frequencies and percentages (%). Differences between continuous variables were evaluated using the Kruskal–Wallis test, while categorical variables were compared using the chi-square test. A *p*-value < 0.05 was considered statistically significant. A univariate analysis was performed to assess the correlations between the change in eGFR (ΔGFR, T2-T0) and the following variables: age, baseline renal function (CKD-EPI T0), and hemoglobin. Spearman’s rank correlation coefficient was used to evaluate the associations due to non-normal distribution of the variables.

The association between predictor variables and the development of AKI was assessed using multivariate logistic regression. The initial multivariate model included the following variables: age, eGFR at baseline, diagnosis of diabetes and immunotherapy. A bidirectional stepwise selection based on AIC was applied to select the most significant variables. Results are reported as odds ratios (ORs) with 95% confidence intervals (CIs). A *p*-value < 0.05 was considered statistically significant.

## 3. Results

Demographic and clinical characteristics of the patients enrolled in this study are summarized in Table 1. Clinical data of 70 patients were analyzed in this study. A total of 42 patients had arterial hypertension, 11 patients had dyslipidemia, 9 patients had a diagnosis of diabetes, and 20 patients had stable cardiovascular diseases and were under follow-up at the time of the study. Median age was 71.5 years and 43 (61.4%) patients were male. The median serum creatinine was 1.14 mg/dL and median eGFR was 58 mL/min. The median hemoglobin was 12.6 g/dL. The most frequent neoplasms were the following: lung cancer (28.6% patients), renal cancer and breast cancer (15.7% patients) and colorectal cancer (11.4% patients). Most of the patients (65.7%) had metastases and were treated as follows: immunotherapy (35.7%), anti-VEGF (18.6%), and cisplatin (2.9%). Forty-three (61.4%) patients have presented AKI and 18 (25.7%) patients showed CKD (Table 1). No significant differences were found in hemoglobin, 24 h protein excretion and blood gas analysis between T0 and T2 when comparing patients undergoing immunotherapy, targeted therapy (anti-VEGF agents) and chemotherapy (platinum-based chemotherapy, cisplatin). Only patients receiving targeted therapy showed a worsening of 24 h protein excretion between T0 and T2, even if not significant (Table 2). Between T0 and T2, 22% of patients showed progression of the oncological pathology that required changes to the oncological treatment. The comparative analysis between the 25 patients treated with immunotherapy and the 45 patients treated with traditional therapies highlighted a statistically significant reduction in renal function in the first group compared to the latter. Indeed, patients treated with immunotherapy showed a statistically significant higher blood urea nitrogen, [85 mg/dLvs. 53 mg/dL, *p* < 0.01] and creatinine [2.07 mg/dL vs. 1.51 mg/dL, *p* < 0.01] compared to patients treated with traditional therapy (Table 3). Moreover, median eGFR was significantly lower in patients treated with immunotherapy compared to patients treated with traditional therapy [31 mL/min vs. 42 mL/min, *p* < 0.01]. Patients treated with immunotherapy were affected mostly by lung cancer (56%), bladder cancer and renal cancer (12%), whilst patients treated with traditional therapy suffered mostly from breast cancer (24.4%), colorectal cancer and renal cancer (17.8%) (*p* < 0.001) (Table 3). Therapy discontinuation was performed in 14 (56%) patients treated with immunotherapy and 7 (19.4%) patients treated with traditional therapy (*p* < 0.01). Three patients died: two patients were in treatment with Pembrolizumab and one patient was in treatment with Atezolizumab. Table 3 shows the comparative analysis between patients treated with immunotherapy and patients treated with traditional therapies. Of the 25 patients in treatment with immunotherapy, 13 (52%) developed irAEs (Table 4). We performed a sub-analysis in the cohort of patients treated with immunotherapy, excluding three patients who died, to evaluate the demographic and clinical differences between patients who developed irAEs (*n* = 13) and patients who did not (*n* = 9). We found no difference between these groups except for the renal damage and for the frequency of discontinuation of the therapy. Indeed, patients who developed irAEs showed mostly AKIs (92.3%), whilst patients who did not developed irAEs showed both AKIs and CKDs (44.4%) (*p* < 0.01). Therapy discontinuation was performed in 84.6% patients with irAEs and in 11.1% patients without irAEs (*p* < 0.001). These results are summarized in Table 4. Among the 13 patients who developed irAEs, most events were grade 1 or 2 (grade 1 toxicities allowed continuation of ICIs with close monitoring, except for selected neurologic, hematologic, or cardiac events; grade 2 toxicities required temporary discontinuation of ICIs and initiation of corticosteroid therapy (prednisone 0.5–1 mg/kg/day or equivalent)). Overall, five patients required systemic corticosteroids for irAE management. No patient required replacement therapy as a result of renal damage from oncological therapy.

**Figure 1:**
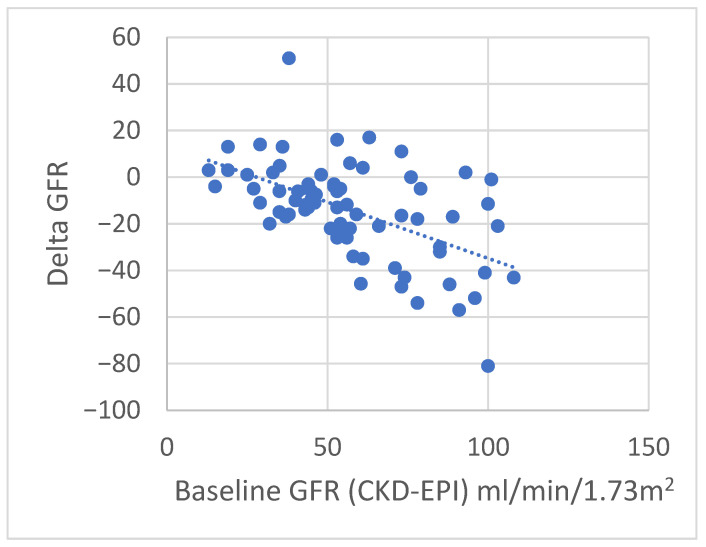
The analysis of linear correlations between baseline renal function (CKD-EPI T0) and the change in GFR (ΔGFR, T4-T0) (rho = −0.51; *p* < 0.001).

The clinical, demographic and haematochemical parameters, the type of primary neoplasm, the presence of metastases, the type of therapy, and the development of irAEs were evaluated according to the recovery or otherwise of renal function after AKI (Table 5). The difference in T0 creatinine levels between the three patient groups was significant, *p* = 0.025, with higher levels observed in those who had full recovery, then in those who had partial recovery, and finally in those who had no recovery of kidney function; the latter group showed a high prevalence of patients with subacute or acute on chronic renal damage compared to the others (*p* = 0.001). The analysis of linear correlations showed that only a significant negative correlation was observed between baseline renal function (CKD-EPI T0) and the change in eGFR (ΔGFR, T2-T0) (rho = −0.51; *p* < 0.001) (Figure 1). No significant correlations were found with age or hemoglobin. In the multivariate logistic regression, the only variable significantly associated with the development of AKI was immunotherapy (OR 2.74, 95% CI 1.05–7.45, *p* = 0.043) (Table 6). Age, baseline renal function, and diabetes were not significantly associated with the outcome and were excluded from the final model through stepwise selection. This indicates that patients receiving immunotherapy had approximately 2.7 times higher odds of developing AKI compared to those who did not receive immunotherapy, independent of the other factors considered.

## 4. Discussion

In our cohort, renal involvement was highly prevalent, with a substantial burden of both AKI and CKD. The observed prevalence of CKD (25.7%) is consistent with that reported in large oncologic cohorts [13], including the IRMA studies [14,15] and other international series [16], confirming that impaired renal function represents a common comorbidity among cancer patients. This finding has relevant clinical implications, as CKD may influence both therapeutic decision-making and patient outcomes. Renal toxicity varied according to the type of oncologic treatment [17,18,19]. In particular, worsening proteinuria was more frequently observed among patients receiving targeted therapies, which is in line with the known effects of these agents on glomerular permeability [20,21]. Moreover, a significantly higher incidence of AKI was observed in patients undergoing immunotherapy. After adjustment for potential confounders, immunotherapy exposure was independently associated with an increased risk of AKI, with approximately a 2.7-fold higher odds compared with patients not receiving immunotherapy. This observation supports the growing recognition of irAEs as a clinically relevant complication of immune checkpoint inhibitor therapy [22]. Overall, our findings highlight the importance of careful renal monitoring in oncologic patients, particularly in those treated with targeted agents or immunotherapy. Early recognition of renal impairment may allow timely interventions, optimization of cancer therapy, and potentially improved outcomes [23]. An important clinical consideration is how renal impairment affects therapeutic decisions and patient outcomes. We found that patients receiving immunotherapy had a significantly higher rate of treatment discontinuation compared with other therapeutic groups, with nearly half developing renal irAEs. Electrolyte abnormalities—frequently associated with immunotherapy—were also present in our cohort, including a significant decrease in serum sodium, although values remained within the normal range. Hyponatremia increases mortality risk in oncologic patients [24], and its correction has been shown to improve survival, as demonstrated in the SALT-1, SALT-2 [25], and EVEREST trials [26]. Hyponatremia may be directly caused by the primary tumor, be secondary to anticancer therapy, or result from comorbidities. The main cause of hyponatremia in this population is SIADH (Syndrome of Inappropriate Antidiuretic Hormone Secretion), which accounts for more than 30% of cases in cancer patients [24]. Among the drugs most frequently associated with SIADH are platinum derivatives, alkylating agents (particularly cyclophosphamide), targeted therapies, immunotherapies, and vinca alkaloids [27], while immunotherapy may also induce hypophysitis [3]. In our study, serum potassium and phosphorus levels did not show significant changes, likely due to compensatory mechanisms at the distal tubule and intestinal levels as well as an adequate dietary regimen, in patients with CKD stage 3–4 and relatively preserved eGFR. Interestingly, although patients with higher baseline renal function experienced a larger absolute decline in eGFR over time, patients who developed AKI while receiving immunotherapy often showed a more favorable clinical course. This apparent discrepancy may reflect prompt recognition and standardized management of renal irAEs, rather than a true functional recovery in patients with lower baseline eGFR. Baseline renal function therefore appears to influence the magnitude of eGFR change, while early diagnosis and appropriate management may mitigate long-term renal damage [27]. Our data underline the need for structured renal surveillance programs in oncology settings, particularly in the era of targeted therapies and immune checkpoint inhibitors. Baseline nephrological assessment, periodic monitoring of kidney function and proteinuria, and early management of electrolyte disorders should be considered integral components of cancer care. A multidisciplinary approach involving oncologists and nephrologists is crucial to balance treatment efficacy with renal safety and to reduce unnecessary therapy discontinuations.

### Study Limitations

This study has several limitations that should be acknowledged.

Firstly, although the design was prospective and longitudinal, the overall sample size was relatively limited, and the number of patients receiving ICIs was small. These factors may have reduced the statistical power of some subgroup comparisons, particularly those involving immunotherapy versus targeted agents. Secondly, being a single-center study, the findings may not be fully generalizable to other oncological or nephrological settings, where cancer types, disease stages, and therapeutic protocols may differ substantially. Thirdly, despite the use of multivariable logistic regression to adjust for relevant covariates, residual confounding cannot be excluded given the clinical heterogeneity of the population. Baseline differences between treatment groups—such as tumor type, disease stage, and prior therapy—could partially account for variations in renal outcomes. Consequently, the comparisons presented herein should be regarded as descriptive and hypothesis-generating rather than inferential. Fourthly, the number of AKI events within specific subgroups was modest, which may have led to partial overfitting of the multivariate models. Thus, the observed associations between immunotherapy and AKI risk should be interpreted with caution and viewed as exploratory. Finally, the follow-up relied on clinically scheduled assessments (T0, T1, and T2); therefore, transient AKI episodes or early renal recovery could have been underestimated. Despite these limitations, this study provides valuable preliminary evidence on the comparative nephrotoxicity of ICIs versus other oncological therapies and underscores the need for larger, multi-center prospective studies to validate and expand upon these findings.

## 5. Conclusions

While immunotherapies and targeted agents have significantly improved cancer outcomes, they are associated with non-negligible renal toxicities. Our study confirms a high burden of both AKI and CKD among cancer patients. In particular, treatment with immunotherapy was independently associated with a higher risk of AKI, highlighting renal irAEs as a relevant clinical complication in this population. Chronic kidney disease was highly prevalent in our cohort, underscoring the importance of baseline renal assessment and close monitoring during oncologic treatment. Early recognition, careful monitoring, and multidisciplinary management are essential to minimize kidney damage while preserving oncologic treatment efficacy, reduce treatment interruptions, and improve overall patient outcomes through personalized therapy.

## Figures and Tables

**Table 1:** Demographic and clinical characteristics of 70 patients enrolled.

Age, years, median (IQR)	71.5 (63–78)
F/M, *n* (%)	27 (38.6)/43 (61.4)
BMI, Kg/m^2^, median (IQR)	24.17 (20.96–29.39)
SBP/DBP, mmHg, median (IQR)	130 (120–140)/70 (70–80)
HR, bpm, median (IQR)	75 (64–84)
Smoker, *n* (%)	
Yes	9 (12.9)
No	32 (45.7)
Previously	29 (41.4)
Blood urea nitrogen, mg/dL, median (IQR)	66 (40–90)
Serum creatinine baseline, mg/dL, median (IQR)	1.14 (1–1.5)
eGFR, mL/min, median (IQR)	58 (44–78)
Na^+^, mmol/L, median (IQR)	140 (137–143)
K^+^, mmol/L, median (IQR)	4.68 (4.2–5.3)
Cl^−^, mmol/L, median (IQR)	104 (101–106)
Ca^2+^, mmol/L, median (IQR)	9.4 (9.1–9.8)
P, mg/dL, median (IQR)	3.6 (3.1–4.1)
Mg, mg/dL, median (IQR)	2 (1.7–2.1)
Serum uric acid, mg/dL, median (IQR)	6.9 (5.2–7.7)
Hemoglobin (g/dL) (IQR)	12.6 (1.2–2.2)
Primary cancer, *n* (%)	
Kidney	11 (15.7)
Colorectal	8 (11.4)
Lung	20 (28.6)
Liver	1 (1.4)
Breast	11 (15.7)
Head–neck	4 (5.7)
Prostate	3 (4.3)
Melanoma	2 (2.9)
Testicle	2 (2.9)
Ovary	1 (1.4)
Pancreas	1 (1.4)
Bladder	3 (4.3)
Stomach	1 (1.4)
Skin	2 (2.9)
Metastasis, *n* (%)	46 (65.7)
Immunotherapy, *n* (%)	25 (35.7)
Anti-VEGF, *n* (%)	
Yes	13 (18.6)
No	54 (77.1)
Previously	3 (4.3)
Cisplatin, *n* (%)	
Yes	2 (2.9)
No	55 (79.7)
Previously	12 (17.4)
Renal damage after treatment *n* (%)	
AKI	43 (61.4)
CKD	18 (25.7)
Other	9 (12.9)
Therapy discontinuation, *n* (%)	21 (30)

IQR: interquartile range; F: female; M: male; BMI: body mass index; SBP: systolic blood pressure; DBP: diastolic blood pressure; HR: heart rate; eGFR: estimated glomerular filtration rate; VEGF: vascular endothelial growth factor; AKI: acute kidney injury; CKD: chronic kidney disease.

**Table 2:** Comparative analysis of 24 h protein excretion between T0 and T2 in all the patients.

	24 h Protein Excretion T0	24 h Protein Excretion T2	*p* Value
Immunotherapy Yes No	375.5 (185–569.5) 290 (214–600)	455 (210–815) 300 (200–1080)	0.815
Anti-VEGF Yes No Previous	195 (57.5–385) 290 (220–639) -	653 (300–1644) 315 (185–900) 220 (200–240)	0.302
Cisplatin Yes No Previous	- 295 (182–550) 245 (220–639)	- 330 (200–900) 220 (220–220)	0.636

VEGF: vascular endothelial growth factor.

**Table 3:** Comparative analysis between 25 patients treated with immunotherapy and 45 patients treated with traditional therapies.

	Traditional Therapy	Immunotherapy	*p*
Age, years, median (IQR)	71 (63–75)	75 (67–79)	NS
F/M, *n* (%)	20 (44.4)/25 (55.6)	7 (28)/18 (72)	NS
BMI, Kg/m^2^, median (IQR)	23.51 (20.96–30.11)	24.27 (22.03–24.56)	NS
SBP/DBP, mmHg, median (IQR)	125 (120–140)/70 (70–80)	130 (127.5–142.5)/80 (70–85)	NS
HR, bpm, median (IQR)	75 (60–85)	77 (65–83)	NS
Smoker, *n* (%)			NS
Yes	3 (6.7)	6 (24)	
No	23 (51.1)	9 (36)	
Previously	19 (42.2)	10 (40)	
Blood urea nitrogen, mg/dL, median (IQR)	53 (31.5–75.6)	85 (70–110)	<0.01
Serum creatinine, mg/dL, median (IQR)	1.51 (1.26–2.2)	2.07 (1.8–2.6)	<0.01
eGFR, mL/min, median (IQR)	42 (27–58)	31 (22–38)	<0.01
Na^+^, mmol/L, median (IQR)	141 (139–143)	139 (135–141)	<0.05
K^+^, mmol/L, median (IQR)	4.6 (4.2–5.15)	4.85 (4.3–5.3)	NS
Cl^−^, mmol/L, median (IQR)	104 (101–106)	104 (101–105)	NS
Ca^2+^, mmol/L, median (IQR)	9.4 (9.1–9.8)	9.3 (9–10)	NS
P, mg/dL, median (IQR)	3.6 (2.9–4.1)	3.45 (3.15–4.03)	NS
Mg, mg/dL, median (IQR)	2 (1.67–2.2)	1.9 (1.72–2)	NS
Serum uric acid, mg/dL, median (IQR)	6.3 (4.7–7.6)	7.25 (6.9–8.9)	NS
Hemoglobin (g/dL)(IQR)	12.1 (1.9–2.1)	11.9 (1.8–2.3)	NS
Primary cancer, *n* (%)			<0.001
Kidney	8 (17.8)	3 (12)	
Colorectal	8 (17.8)	0 (0)	
Lung	6 (13.3)	14 (56)	
Liver	1 (2.2)	0 (0)	
Breast	11 (24.4)	0 (0)	
Head–neck	2 (4.4)	2 (8)	
Prostate	3 (6.7)	0 (0)	
Melanoma	0 (0)	2 (8)	
Testicle	2 (4.4)	0 (0)	
Ovary	1 (2.2)	0 (0)	
Pancreas	1 (2.2)	0 (0)	
Bladder	0 (0)	3 (12)	
Stomach	0 (0)	1 (4)	
Skin	2 (4.4)	0 (0)	
Metastasis, *n* (%)	28 (62.2)	18 (72)	NS
Renal damage after treatment, *n* (%)			NS
AKI	24 (53.3)	19 (76)	
CKD	13 (28.9)	5 (20)	
Other	8 (17.8)	1 (4)	
Therapy discontinuation, *n* (%)	7 (19.4)	14 (56)	<0.01
Exitus ^†^, *n* (%)	0 (0)	3 (12)	<0.05

^†^ Two patients were in treatment with Pembrolizumab and one patient was in treatment with Atezoliz; IQR: interquartile range; F: female; M: male; BMI: body mass index; SBP: systolic blood pressure; DBP: diastolic blood pressure; HR: heart rate; eGFR: estimated glomerular filtration rate; AKI: acute kidney injury; CKD: chronic kidney disease. NS: Not Significant.

**Table 4:** Comparative analysis between 13 patients with immunotherapy-related adverse events (irAEs) and 9 patients without irAE in the cohort of patients treated with immunotherapy.

	Not irAEs	irAEs	*p*
Age, years, median (IQR)	78 (71–82)	75 (64–78)	NS
F/M, *n* (%)	0 (0)/9 (100)	6 (46.2)/7 (53.8)	NS
BMI, Kg/m^2^, median (IQR)	24.21 (24.17–24.24)	22.09 (18.43–28.56)	NS
SBP/DBP, mmHg, median (IQR)	130 (130–140)/80 (70–80)	130 (125–160)/80 (70–90)	NS
HR, bpm, median (IQR)	78 (70–89.5)	73 (65–83)	NS
Smoker, *n* (%)			NS
Yes	2 (22.2)	4 (30.8)	
No	3 (33.3)	3 (23.1)	
Previously	4 (44.4)	6 (46.2)	
Blood urea nitrogen, mg/dL, median (IQR)	74 (49.5–86)	87.5 (74–110.5)	NS
Serum creatinine, mg/dL, median (IQR)	1.89 (1.8–2.51)	2.23 (1.68–2.58)	NS
eGFR, mL/min, median (IQR)	36 (25–45)	29 (22–34)	NS
Na^+^, mmol/L, median (IQR)	137 (134–140)	139 (135–141)	NS
K^+^, mmol/L, median (IQR)	4.95 (4.4–5.3)	4.7 (4.1–5.45)	NS
Cl^−^, mmol/L, median (IQR)	103 (102–104)	103 (100.5–107.5)	NS
Ca^2+^, mmol/L, median (IQR)	9.8 (9.7–10)	9.2 (9.1–9.6)	NS
P, mg/dL, median (IQR)	3.2 (2.8–3.6)	3.3 (3.2–3.72)	NS
Mg, mg/dL, median (IQR)	2.05 (2–2.1)	1.73 (1.7–1.8)	NS
Serum uric acid, mg/dL, median (IQR)	7.3 (7.2–7.7)	7.9 (5.4–9.1)	NS
Primitive cancer, *n* (%)			NS
Kidney	1 (11.1)	2 (15.4)	
Lung	4 (44.4)	8 (61.5)	
Head–neck	1 (11.1)	1 (7.7)	
Melanoma	1 (11.1)	1 (7.7)	
Bladder	2 (22.2)	1 (7.7)	
Metastasis, *n* (%)	7 (77.8)	9 (69.2)	NS
Renal damage after treatment *n* (%)			<0.01
AKI	4 (44.4)	12 (92.3)	
CKD	4 (44.4)	0 (0)	
Other	1 (11.2)	1 (7.7)	
Therapy discontinuation, *n* (%)	1 (11.1)	11 (84.6)	<0.001

IQR: interquartile range; F: female; M: male; BMI: body mass index; SBP: systolic blood pressure; DBP: diastolic blood pressure; HR: heart rate; eGFR: estimated glomerular filtration rate; AKI: acute kidney injury; CKD: chronic kidney disease. NS: Not Significant.

**Table 5:** Comparative analysis in the cohort of 33 patients with acute kidney injury (AKI) according to renal function recovery (total, partial or absent).

	Total Recovery (*n* = 7)	Partial Recovery (*n* = 9)	No Recovery (*n* = 17)	*p*
Age, years, median (IQR)	75 (58–78)	75 (73–85)	71 (63–78)	NS
F/M, *n* (%)	4 (57.1)/3 (42.9)	4 (44.4)/5 (55.6)	7 (41.2)/10 (58.8)	NS
BMI, Kg/m^2^, median (IQR)	29.39 (24.17–32.81)	24.34 (24.34–24.34)	23.91 (20.96–30.37)	NS
SBP/DBP, mmHg, median (IQR)	130 (105–130)/75 (60–80)	122.5 (120–137.5)/70 (70–75)	130 (120–130)/75 (70–85)	NS
HR, bpm, median (IQR)	64 (61–65)	83 (83–83)	75 (67–96)	NS
Smoker, *n* (%)				NS
Yes	1 (14.3)	0 (0)	2 (11.8)	
No	1 (14.3)	6 (66.7)	5 (29.4)	
Previously	5 (71.4)	3 (33.3)	10 (58.8)	
Blood urea nitrogen, mg/dL, median (IQR)	85 (59–91)	78 (55–104.9)	54 (49–77)	NS
Serum creatinine, mg/dL, median (IQR)	2.51 (1.8–2.6)	2.37 (2.24–3.81)	1.72 (1.33–2.12)	<0.05
eGFR, mL/min, median (IQR)	25 (18–48)	19 (15–29)	38 (29–53)	<0.05
Na^+^, mmol/L, median (IQR)	139 (132–141)	141 (135–143)	139 (135–143)	NS
K^+^, mmol/L, median (IQR)	5.3 (4.3–5.8)	4.9 (4.2–5.3)	4.6 (4.2–4.8)	NS
Cl^−^, mmol/L, median (IQR)	101 (94–102)	105.5 (105–106)	106 (105–110)	NS
Ca^2+^, mmol/L, median (IQR)	9.45 (5.78–9.9)	9.3 (9.15–9.5)	9.6 (9.2–9.8)	NS
P, mg/dL, median (IQR)	3.57 (2.8–4.34)	4.6 (4.6–4.6)	3.3 (2.9–3.4)	NS
Mg, mg/dL, median (IQR)	1.7 (1.7–1.7)	1.44 (1.44–1.44)	1.85 (1.72–2.1)	NS
Serum uric acid, mg/dL, median (IQR)	6.9 (3.9–7.7)	-	7.05 (6.6–7.3)	NS
Primary cancer, *n* (%)				NS
Kidney	1 (14.3)	1 (11.1)	1 (5.9)	
Colorectal	0 (0)	1 (11.1)	3 (17.6)	
Lung	5 (71.4)	3 (33.3)	4 (23.5)	
Liver	0 (0)	0 (0)	1 (5.9)	
Breast	0 (0)	2 (22.2)	4 (23.5)	
Head–neck	0 (0)	0 (0)	1 (5.9)	
Prostate	0 (0)	2 (22.2)	0 (0)	
Melanoma	0 (0)	0 (0)	1 (5.9)	
Testicle	1 (14.3)	0 (0)	0 (0)	
Bladder	0 (0)	0 (0)	2 (11.8)	
Metastasis, *n* (%)	3 (42.9)	6 (66.7)	13 (76.5)	NS
Immunotherapy, *n* (%)	5 (71.4)	3 (33.3)	6 (35.3)	NS
Anti-VEGF, *n* (%)	0 (0)	1 (11.1)	2 (11.8)	NS
Cisplatin, *n* (%)	1 (14.3)	0 (0)	1 (5.9)	NS
Renal damage *n* (%)				<0.001
AKI	3 (42.9)	3 (33.3)	0 (0)	
AKI in CKD	4 (57.1)	4 (44.4)	6 (35.3)	
Subacute AKI	0 (0)	2 (22.2)	11 (64.7)	

IQR: interquartile range; F: female; M: male; BMI: body mass index; SBP: systolic blood pressure; DBP: diastolic blood pressure; HR: heart rate; eGFR: estimated glomerular filtration rate; VEGF: vascular endothelial growth factor; AKI: acute kidney injury. NS: Not Significant.

**Table 6:** Multivariate logistic regression, the only variable significantly associated with the development of AKI was immunotherapy (OR 2.74, 95% CI 1.05–7.45, *p* = 0.043).

Variable	Coefficient (β)	Std. Error	*p*-Value	Odds Ratio (OR)	95% CI OR
Intercept	−0.496	0.339	0.143	0.61	0.31–1.17
Immunotherapy	1.007	0.498	0.043	2.74	1.05–7.45

## Data Availability

The data presented in this study are available on request from the corresponding author. The data are not publicly available due to privacy and ethical reasons.

## References

[1] Global Burden of Disease 2019 Cancer Collaboration (2022). Cancer Incidence, Mortality, Years of Life Lost, Years Lived with Disability, and Disability-Adjusted Life Years for 29 Cancer Groups from 2010 to 2019: A Systematic Analysis for the Global Burden of Disease Study 2019. JAMA Oncol..

[2] de Francisco A.L., Macía M., Alonso F., García P., Gutierrez E., Quintana L.F., Quiroga B., Torregrosa I. (2019). Onco-Nephrology: Cancer, chemotherapy and kidney. Nefrologia.

[3] Jagieła J., Bartnicki P., Rysz J. (2021). Nephrotoxicity as a Complication of Chemotherapy and Immunotherapy in the Treatment of Colorectal Cancer, Melanoma and Non-Small Cell Lung Cancer. Int. J. Mol. Sci..

[4] Herrmann S.M., Perazella M.A. (2020). Immune Checkpoint Inhibitors and Immune-Related Adverse Renal Events. Kidney Int. Rep..

[5] Haanen J., Obeid M., Spain L., Carbonnel F., Wang Y., Robert C., Lyon A.R., Wick W., Kostine M., Peters S. (2022). Management of toxicities from immunotherapy: ESMO Clinical Practice Guideline for diagnosis, treatment and follow-up. Ann. Oncol..

[6] Izzedine H. (2020). Renal toxicities of targeted therapies in oncology. Nephrol. Ther..

[7] Fofi C., Festuccia F. (2021). Onconephrology: A New Challenge for the Nephrologist. Contrib. Nephrol..

[8] Schanz M., Schricker S., Pfister F., Alscher M.D., Kimmel M. (2018). Renal complications of cancer therapies. Drugs Today.

[9] Levin A., Stevens P.E., Bilous R.W., Coresh J., De Francisco A.L., De Jong P.E., Griffith K.E., Hemmelgarn B.R., Iseki K., Lamb E.J. (2024). Kidney Disease: Improving Global Outcomes (KDIGO) CKD Work Group. KDIGO 2024 Clinical Practice Guideline for the Evaluation and Management of Chronic Kidney Disease. Kidney Int..

[10] Kellum J.A., Lameire N., KDIGO AKI Guideline Work Group (2013). Diagnosis, evaluation, and management of acute kidney injury: A KDIGO summary (Part 1). Crit. Care.

[11] Levey A.S. (2022). Defining AKD: The Spectrum of AKI, AKD, and CKD. Nephron.

[12] Patschan D., Stasche F., Erfurt S., Matyukhin I., Ritter O., Safi W. (2025). Recovery of kidney function in acute kidney injury. J. Nephrol..

[13] Canter D., Kutikov A., Sirohi M., Street R., Viterbo R., Chen D.Y., Greenberg R.E., Uzzo R.G. (2011). Prevalence of baseline chronic kidney disease in patients presenting with solid renal tumors. Urology.

[14] Launay-Vacher V. (2010). Epidemiology of chronic kidney disease in cancer patients: Lessons from the IRMA study group. Semin. Nephrol..

[15] Launay-Vacher V., Oudard S., Janus N., Gligorov J., Pourrat X., Rixe O., Morere J.F., Beuzeboc P., Deray G., Renal Insufficiency and Cancer Medications (IRMA) Study Group (2007). Prevalence of renal insufficiency in cancer patients and implications for anticancer drug management: The renal insufficiency and anticancer medications (IRMA) study. Cancer.

[16] Janus N., Launay-Vacher V., Byloos E., Machiels J.-P., Duck L., Kerger J., Wynendaele W., Canon J.-L., Lybaert W., Nortier J. (2010). Cancer and renal insufficiency results of the BIRMA study. Br. J. Cancer.

[17] García-Carro C., Draibe J., Soler M.J. (2023). Onconephrology: Update in Anticancer Drug-Related Nephrotoxicity. Nephron.

[18] Yarandi N., Shirali A.C. (2023). Onconephrology: Core Curriculum 2023. Am. J. Kidney Dis..

[19] Lameire N.H., Flombaum C.D., Moreau D., Ronco C. (2005). Acute renal failure in cancer patients. Ann. Med..

[20] Lai S., Amabile M.I., Mazzaferro S., Mitterhofer A.P., Mazzarella A., Galani A., Imbimbo G., Cianci R., Pasquali M., Molfino A. (2020). Effects of sunitinib on endothelial dysfunction, metabolic changes, and cardiovascular risk indices in renal cell carcinoma. Cancer Med..

[21] Lai S., Molfino A., Seminara P., Longo F., Innico G., Coppola B., Mastroluca D., Galani A., Dimko M., Aceto P. (2018). Vascular Endothelial Growth Factor Inhibitor Therapy and Cardiovascular and Renal Damage in Renal Cell Carcinoma. Curr. Vasc. Pharmacol..

[22] Shaikh A. (2022). Immunotherapies and renal injury. Curr. Opin. Toxicol..

[23] Ishii T., Fujimaru T., Nakano E., Takahashi O., Nakayama M., Yamauchi T., Komatsu Y. (2020). Association between chronic kidney disease and mortality in stage IV cancer. Int. J. Clin. Oncol..

[24] Florio G., Iervolino A., Simeoni M., Perna A.F., Trepiccione F. (2023). Iposodiemia e disordini elettrolitici nel paziente oncologico. G Ital. Nefrol..

[25] Schrier R.W., Gross P., Gheorghiade M., Berl T., Verbalis J.G., Czerwiec F.S., Orlandi C., SALT Investigators (2006). Tolvaptan, a selective oral vasopressin V2-receptor antagonist, for hyponatremia. Clinical Trial. N. Engl. J. Med..

[26] Konstam M.A., Gheorghiade M., Burnett J.C., Grinfeld L., Maggioni A.P., Swedberg K., Udelson J.E., Zannad F., Cook T., Ouyang J. (2007). Efficacy of Vasopressin Antagonism in Heart Failure Outcome Study with Tolvaptan (EVEREST) Investigators Effects of oral tolvaptan in patients hospitalized for worsening heart failure: The EVEREST Outcome Trial. Randomized Controlled Trial. JAMA.

[27] Hu M., Wang Q., Liu B., Ma Q., Zhang T., Huang T., Lv Z., Wang R. (2022). Chronic Kidney Disease and Cancer: Inter-Relationships and Mechanisms. Front. Cell Dev. Biol..

